# SAM for Road Object Segmentation: Promising but Challenging

**DOI:** 10.3390/jimaging11060189

**Published:** 2025-06-10

**Authors:** Alaa Atallah Almazroey, Salma kammoun Jarraya, Reem Alnanih

**Affiliations:** Faculty of Computing and Information Technology, Computer Science Department, King Abdulaziz University, Jeddah 21589, Saudi Arabia; smohamad1@kau.edu.sa (S.k.J.); ralnanih@kau.edu.sa (R.A.)

**Keywords:** foundation model, road object segmentation, Segment Anything Model (SAM), autonomous vehicle, artificial intelligent

## Abstract

Road object segmentation is crucial for autonomous driving, as it enables vehicles to perceive their surroundings. While deep learning models show promise, their generalization across diverse road conditions, weather variations, and lighting changes remains challenging. Different approaches have been proposed to address this limitation. However, these models often struggle with the varying appearance of road objects under diverse environmental conditions. Foundation models such as the Segment Anything Model (SAM) offer a potential avenue for improved generalization in complex visual tasks. Thus, this study presents a pioneering comprehensive evaluation of the SAM for zero-shot road object segmentation, without explicit prompts. This study aimed to determine the inherent capabilities and limitations of the SAM in accurately segmenting a variety of road objects under the diverse and challenging environmental conditions encountered in real-world autonomous driving scenarios. We assessed the SAM’s performance on the KITTI, BDD100K, and Mapillary Vistas datasets, encompassing a wide range of environmental conditions. Using a variety of established evaluation metrics, our analysis revealed the SAM’s capabilities and limitations in accurately segmenting various road objects, particularly highlighting challenges posed by dynamic environments, illumination changes, and occlusions. These findings provide valuable insights for researchers and developers seeking to enhance the robustness of foundation models such as the SAM in complex road environments, guiding future efforts to improve perception systems for autonomous driving.

## 1. Introduction

In autonomous driving applications, road object segmentation is a fundamental task that enables self-driving vehicles to perceive and understand their surroundings, ensuring safe and efficient navigation [1]. Accurate segmentation is essential for autonomous vehicles to operate and navigate without the need for human interaction in dynamic driving environments [2]. As autonomous driving technology advances, the demand for robust and reliable segmentation models will increase, as road object segmentation is considered a basis for other autonomous driving tasks, such as road object classification, recognition, and tracking [3]. However, autonomous vehicles still require an accurate segmentation model as the current state of road object segmentation technology is not ideal, and autonomous vehicle perception systems continue to suffer from limitations such as sensor constraints, resolution, susceptibility to adverse weather conditions (e.g., rain and fog), occlusions, and variations in illumination conditions [4], all of which complicate the segmentation process and compromise the accuracy and reliability of captured road information.

Most of the previously proposed models for image segmentation rely on deep learning techniques [5,6,7] and were often trained and tested in specific scene environments with limited variability. These models may demonstrate strong performance in specific scenarios but often struggle to generalize to diverse road conditions, weather variations, and lighting changes. Moreover, they may be limited in their ability to handle the wide range of object types and road environments encountered in real-world driving scenarios. This inherent limitation of traditional supervised approaches poses significant challenges for autonomous driving systems, which demand highly robust and adaptable object perception in constantly evolving environments.

Efforts to eliminate the limitations of existing models have led to the development of Large Vision Models (LVMs), which represent a big leap for computer vision tasks. These models are specially developed to tackle complicated visual tasks by employing deep neural network architectures trained on large-scale datasets. These large models, called foundation models, serve as the building blocks for various vision tasks and provide a solid starting point for further advancements [8]. One of the notable foundation models in computer vision is the Segment Anything Model (SAM), which is considered the first foundation model for image segmentation [9]. The SAM foundation model was trained on a large-scale dataset, enabling powerful zero-shot transfer learning and flexible prompting. The SAM has demonstrated strong performance in a wide range of segmentation tasks, and it does not require retraining for specific tasks; instead, it adapts swiftly to a new downstream task [9].

Despite its advantages, the SAM’s performance in road object segmentation remains largely unexplored. As the SAM has been pretrained on general image datasets, its generalization ability may be limited when applied to specific data sources, such as road object data under varying conditions, including weather, illumination, or crowd density. The SAM has been evaluated in multiple domains, including medical [10,11], urban planning [12], and mobility infrastructure [13]; each domain presents unique challenges that can affect its accuracy. Given the unique challenges of road object segmentation, such as the need to adapt to changing environments, including dynamic road environments, illumination, and occlusion, it is essential to evaluate the SAM’s ability to handle these tasks effectively. With the SAM’s unprecedented generalization capabilities and its potential to circumvent the extensive retraining burden of traditional models, understanding its direct applicability to the complex and safety-critical domain of road object segmentation is paramount. Consequently, its inherent zero-shot generalization for diverse road object segmentation remains largely unexplored in a comprehensive manner, and the unique challenges it faces in this domain are not yet well defined, representing a critical gap in assessing its immediate utility and limitations for autonomous perception.

Researchers have begun to explore the SAM’s performance in autonomous driving contexts; for instance, the authors of [14] investigated the SAM’s performance in self-driving tasks under specific adverse weather conditions. However, our study distinguishes itself from previous work by providing a comprehensive zero-shot evaluation across a wider array of diverse road scenes and varying environmental conditions (beyond just specific weather types, e.g., varied illumination, crowd density, and geographical locations). Crucially, we also explicitly define and characterize the specific challenges that the SAM faces in road object segmentation in this unprompted configuration, an aspect not thoroughly detailed in prior evaluations such as [14]. This foundational zero-shot analysis offers unique insights into the SAM’s intrinsic capabilities before any specialized adaptations. While domain-specific adaptations of the SAM, such as MedSAM [15] and GeoSAM [13], have been proposed for other domains, this study focused on evaluating the SAM in a zero-shot setting without explicit prompts in order to establish a clear performance baseline for road object segmentation without task-specific enhancements. We aimed to fill a key research gap by comprehensively evaluating the SAM’s performance in the domain of road object segmentation and by rigorously identifying the challenges it faces in diverse road scenarios. We utilized the “Segment Everything” mode, which allows the SAM to automatically generate masks for all potential objects in a scene, without requiring specific object prompts. This approach is particularly suitable for dynamic environments such as road scenes, where the diversity of objects and constant changes make prompt-based segmentation impractical. We thoroughly evaluated the SAM on the KITTI [16], BDD100K [17], and Mapillary Vistas [18] datasets with varying weather, illumination, and complex road conditions. This evaluation involved a quantitative analysis using standard segmentation metrics alongside qualitative analysis to identify specific challenges. We believe that our findings will be valuable to researchers and developers working on autonomous driving and other road scene understanding applications, guiding future research and development efforts to improve the SAM’s performance and robustness in complex road environments.

The contributions of this study are summarized as follows:

- This study presents the pioneering comprehensive evaluation of the Segment Anything Model (SAM) specifically for road object segmentation, with an assessment of its performance across diverse road environmental conditions (occlusion, illumination variations, weather conditions).
- This work highlights the specific challenges faced by the SAM in achieving the effective segmentation of road objects under these various environmental conditions, underscoring limitations crucial for its applicability in autonomous driving scenarios.
- A thorough quantitative evaluation was conducted using five established evaluation metrics—Mean Intersection over Union (mIoU), Recall, Precision, F1 Score, and Highest IoU—to provide a robust understanding of the SAM’s strengths and weaknesses, complemented by qualitative evidence for visualizing these segmentation challenges.
- The SAM’s generalizability and reliability were verified by evaluating it on three publicly available road object datasets that encompass a wide range of real-world road scenarios: KITTI, BDD100K, and Mapillary Vistas.
- Valuable insights are provided by identifying specific challenges and providing a detailed quantitative analysis, which can guide future research toward targeted enhancements to improve the accuracy and robustness of the SAM in this critical domain.

The remainder of this paper is organized as follows: Section 2 provides a review of related work on image segmentation. Section 3 details the methodology employed for evaluating the SAM in road object segmentation, encompassing the datasets and experimental setup. Section 4 presents the quantitative and qualitative results obtained from our comprehensive evaluation, and Section 5 discusses the challenges encountered by the SAM in road object segmentation and suggests recommendations for future research. Finally, Section 6 concludes the paper.

## 2. Related Work

Image segmentation is one of the most fundamental research areas in computer vision. It has consistently attracted significant attention from researchers, as it serves as the foundation for pattern recognition and image understanding [3]. Various methods have been developed for image segmentation, with these approaches evolving and improving over time. Traditional segmentation techniques primarily focused on processing individual images, with notable examples including edge detection [19], graph theory [20], and clustering [21]. However, these early methods had several limitations, such as sensitivity to noise, the need for manual parameter tuning, difficulties in extracting high-level semantic information, and challenges in handling complex images. These limitations motivated a significant shift toward deep learning approaches.

Over the past several years, deep learning approaches, particularly Convolutional Neural Networks (CNNs), have been widely applied to overcome the limitations of traditional methods in image segmentation. The Fully Convolutional Network (FCN) proposed by Long et al. [22] is considered a pioneering work that enabled pixel-level classification for semantic segmentation by converting classification probabilities into spatial feature maps. FCNs modify standard CNNs by replacing fully connected layers with convolutional layers [22]. Numerous advances building upon the FCN architecture have been made to better understand spatial relationships and extract features at different levels of detail, aiming for more accurate and efficient segmentation in complex visual scenes. For example, Convolutional Autoencoders (CAEs) [5,6] have often been employed for more efficient dimensionality reduction, making models more suitable for real-time applications. The authors of [6] proposed a hybrid model that combined PSPNet and EfficientNet to create a super-light architecture.

Beyond these initial efforts to increase efficiency, significant advancements have also focused on robust multi-scale feature representation to handle objects of varying sizes in different contexts by using Feature Pyramid Networks (FPNs) to extract multi-scale features for better segmentation results. FPNs achieve this by combining high-level semantic information with low-level detailed features to construct strong semantic features at different scales. For instance, in [23], multiple predictions at different resolutions were proposed to enhance the segmentation of small objects and narrow structures. Similarly, MPNet [24] contains a multi-scale prediction module where pairs of adjacent features are combined for three parallel predictions, with an integrated Atrous Spatial Pyramid Pooling (ASPP) module providing further multi-scale contextual information. Other architectural advancements, such as RGPNet [25], proposed an asymmetric encoder–decoder structure with an adapter module to refine features and improve gradient flow. The authors of [5] applied CAEs in conjunction with FPNs to extract hierarchical features from images and also incorporated a bottleneck residual network to reduce computational complexity. Building upon such efforts to improve computational efficiency for resource-constrained environments, various lightweight architectures have emerged. These include the Shared Feature Reuse Segmentation (SFRSeg) model [26], which focuses on shared feature reuse and context mining. Similarly, in [27], the authors tackle the challenge of real-time performance by proposing an efficient Short-Term Dense Bottleneck (SDB) module and a parallel shallow branch for precise localization. AGLNet [28], in turn, is based on a lightweight encoder–decoder architecture that specifically aims for efficiency while also incorporating a multi-scale context through attention mechanisms.

Another significant direction in semantic segmentation is the use of dilated convolutions in the DeepLab series and its variants to enlarge the receptive field while maintaining spatial resolution. For example, DeepLabV3+ was integrated with the rapid shift superpixel segmentation technique in [2]. Moreover, the authors of [7] modified the DeepLabV3+ framework by incorporating ASPP for more effective multi-scale feature extraction. More recently, attention mechanisms have also been explored to enhance segmentation performance, as seen in DCN-Deeplabv3+ [29], which improves semantic segmentation by involving dual-attention modules based on the Deeplabv3+ network.

Despite the significant progress achieved by deep learning models in image segmentation, inherent limitations persist. A major challenge lies in the fact that most existing approaches are trained and tested on the same dataset, severely restricting their ability to generalize effectively across diverse environments and scenarios. Furthermore, these segmentation models are typically constrained by dataset-specific learning and predefined classes, which limits their adaptability to novel or unseen object categories. Table 1 summarizes the performance of various state-of-the-art models that use deep learning approaches for semantic segmentation benchmarks, illustrating current capabilities and inherent challenges in generalization across diverse environments.

Through efforts to overcome these challenges and enhance generalization, the field has witnessed the emergence of Large Vision Models (LVMs), leveraging extensive training on massive datasets to learn more broadly applicable visual representations. A notable LVM that has gained significant prominence, particularly in image segmentation due to its training on a vast and diverse image collection, is the Segment Anything Model (SAM) [9]. Introduced as the first foundation model specifically for image segmentation, the SAM has an exceptional ability to generate precise segmentation masks for natural images and provides a flexible zero-shot segmentation framework. Numerous studies have been conducted to evaluate the SAM’s performance as an initial step in identifying its strengths and limitations in various domains, including medical imaging [30,31], urban environments [12], and remote scene imagery [32].

In the medical domain, multiple studies have evaluated the zero-shot segmentation performance of the SAM in various tasks and image modalities [30,31,32,33,34]. For instance, the authors of [30] assessed the SAM’s ability to segment polyps in colonoscopy images and found that it struggled to accurately delineate the often-blurred boundaries between polyps and surrounding tissues. Similarly, the researchers in [33] evaluated the SAM in segmenting medical lesions and other different modalities, and they concluded that its segmentation results did not surpass those of state-of-the-art methods in these areas. To further broaden the evaluation, the authors of [31,34] adopted a broader approach, assessing the SAM’s performance with diverse medical image modalities (MRI, CT, X-ray, ultrasound, PET) and various anatomical structures using different prompting strategies. Meanwhile, in [34], the authors constructed a large, multi-modal medical image dataset encompassing 84 object categories to thoroughly assess the SAM’s strengths and weaknesses with different modalities. Moreover, the researchers in [32] evaluated the SAM on various image types, including medical images, remote scene images, and RGB images, focusing on the relationship between perturbations and model vulnerabilities.

Beyond the medical domain, several other studies have also evaluated the SAM’s segmentation performance in other specialized areas, with remote sensing (RS) imagery analysis being a prominent area. For instance, the researchers in [35] evaluated the SAM’s segmentation performance with different remote sensing modalities (airborne images and satellite images) at varying resolutions. Their observations indicated that, while the SAM’s performance shows promise, it is significantly influenced by the applied prompt and the spatial resolution of the imagery. Lower-resolution images presented a challenge for the SAM in accurately defining boundaries between adjacent objects. Similarly, the authors of [12] proposed using the SAM to delineate green areas in urban and agricultural landscapes from RS images. The model encountered several challenges, notably difficulties in precisely segmenting urban green belts and complex urban environments characterized by a mixture of various features. Furthermore, the study suggested that large-sized RS images presented computational challenges, potentially due to the overwhelming amount of feature information.

Whereas the aforementioned studies focused on medical and remote sensing imagery, other research has explored the SAM’s capabilities using standard RGB images. For example, in [36], the authors assessed the SAM for traffic sign segmentation, comparing it against Mask R-CNN using diverse datasets and training data amounts. Their findings indicated that while the SAM did not surpass Mask R-CNN in accuracy, a modified SAM incorporating convolutional layers demonstrated improved generalization. The research also explored the influence of different decoders and analyzed instance-level segmentation, underscoring the SAM’s potential for efficient adaptation in autonomous driving applications.

Consequently, the collective results from these evaluations in various domains indicate an inconsistency in the performance of the SAM. It performs well in certain image modalities or with some objects while struggling with others. This variability appears to be influenced by factors such as the type of chosen prompt, object size, the clarity of boundaries, image complexity, intensity differences, and image resolution. Overall, the aforementioned studies demonstrate that applying the SAM directly in different domains has revealed limitations due to visual differences between these domain-specific images and the natural images on which the SAM was trained. Notably, despite the extensive evaluation of the SAM in domains such as medical imaging and remote sensing, to the best of our knowledge, there is a lack of specific studies dedicated to evaluating the SAM’s performance for road object segmentation under diverse real-world conditions. Based on the results of evaluations in other domains, several studies have focused on adapting the SAM to address these challenges and enhance its performance, aiming to improve its ability to handle domain-specific complexities and its robustness in difficult segmentation tasks.

Numerous studies in the medical domain have attempted to adapt the SAM to enhance its segmentation performance for diverse image modalities. For instance, notable work in this direction is presented in [10,15], with a researcher in [15] introducing the MedSAM model, which was adapted by fine-tuning the SAM on a large-scale medical image segmentation dataset containing 1,570,263 image–mask pairs in 10 imaging modalities and with over 30 cancer types. Although MedSAM demonstrated improved performance over the original SAM, challenges remained, particularly in segmenting vessel-like branching structures, where the suggestions of the bounding box were ambiguous. Similarly, the study in [10] proposed SAM-Med2D, an adapted version of the SAM tailored for 2D medical image segmentation. This adaptation involved fine-tuning the SAM using a large dataset of 4.6 million medical images and 19.7 million corresponding masks. To integrate medical domain knowledge without retraining the entire model, adapter modules were added to each transformer block of the image encoder. This adaptation technique improved the SAM’s performance and enabled it to generalize better across a variety of medical imaging modalities.

Despite the promise of these adaptations, many still rely heavily on prompt engineering to define Regions of Interest (ROIs), which can be labor-intensive and costly, particularly when working with large datasets and multiple classes. To address this challenge, several studies have explored auto-prompting mechanisms aimed at enhancing the SAM’s performance. In [11], the researchers integrated YOLOv8 with the SAM and HQ-SAM to enhance ROI segmentation using different medical imaging modalities. YOLOv8 is effective in identifying bounding boxes around ROIs, while the SAM offers advantages in handling ambiguous regions and providing real-time mask estimates. The integration of YOLOv8 with the SAM and HQ-SAM improves both segmentation accuracy and efficiency. Meanwhile, in [37], the Uncertainty Rectified Segment Anything Model (UR-SAM) framework was proposed to enhance the robustness and reliability of auto-prompting. This approach utilizes prompt augmentation to generate multiple bounding box prompts, producing varied segmentation output. The framework then estimates segmentation uncertainty and applies uncertainty rectification to improve model performance, particularly in challenging medical image segmentation tasks.

In the domain of remote sensing imagery, adaptations of the SAM have been made to tackle the unique challenges associated with geographical segmentation. For example, GeoSAM [13] was developed to enhance the SAM’s segmentation capabilities for mobility infrastructure such as sidewalks and roads in geographical images. The SAM faced difficulties in segmenting these objects due to issues such as texture blending, narrow features, and occlusions. To overcome these challenges, GeoSAM adapts the mask decoder using parameter-efficient techniques, resulting in more accurate segmentation of mobility infrastructure without the need for human intervention. Similarly, in [38], the authors employed a fine-tuning strategy for the SAM to improve the segmentation of water bodies, such as lakes and rivers, in RS images. This approach eliminated the need for a prompt encoder, reducing human interaction, by adding learned embeddings to each image feature, which were then supplied to the mask decoder to predict low-resolution masks. As a result of this adaptation, the SAM’s performance was improved compared to deep learning approaches, but with higher computational costs.

Based on a review of the existing literature, it is evident that, while numerous studies have evaluated and adapted the SAM for various domains, its performance in segmenting road objects, particularly under the diverse and dynamic conditions typical of road environments, remains unclear. In a previous study [14], initial attempts were made to apply the SAM in the autonomous driving domain under different weather conditions, including rain, snow, and fog. However, the authors did not perform a comprehensive analysis of how the SAM handles segmenting road objects when subjected to different factors, such as different illumination levels, different types of roads, and occlusions. Consequently, the specific challenges that the SAM encounters in accurately segmenting road objects in real-world scenarios are still largely undefined and require further exploration. In this study, we aimed to directly address this critical gap by conducting a comprehensive evaluation of the SAM’s ability to segment road objects under complex, real-world conditions. We assessed its performance in various challenging scenarios, meticulously identifying its strengths and limitations in handling the intricacies inherent in road environments.

## 3. Method

This section outlines the method and setups used to evaluate the SAM’s robustness for road object segmentation under diverse conditions. This section starts with a brief overview of the Segment Anything Model (SAM), including the dataset on which it was trained and its architecture, followed by a description of the datasets that were used in this study. Finally, we detail our experimental setup and the evaluation metrics employed to assess the SAM’s performance.

### 3.1. Model Overview

The Segment Anything Model (SAM) is the first foundation model developed for image segmentation tasks [9]. The SAM’s introduction has led to a transformative shift within the computer vision community. This pioneering model has revolutionized how computers perceive and interpret visual information, mirroring how human eyes extract meaningful insights from the visual environment [39]. The SAM was trained on the SA-1B dataset, which contains over 1 billion masks from around 11 million licensed images [9]. The inclusion of a comprehensive dataset allows the SAM to demonstrate remarkable zero-shot generalization, which means it can successfully handle tasks and datasets that it has not been specifically trained on in various image segmentation applications [9]. By leveraging the knowledge acquired through its training on the SA-1B dataset, the SAM establishes a strong foundation for developing more specialized and advanced models in the field of image segmentation.

**Figure 1:**
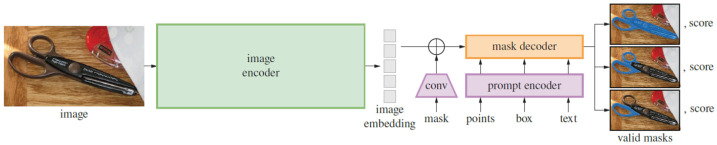
Segment Anything Model (SAM) architecture [9].

The SAM architecture is built upon three essential components, as illustrated in Figure 1 [9]. The first component is the image encoder, which utilizes a vision transformer (ViT) [40] pretrained with Masked Autoencoders (MAEs) [41]; the encoder takes an image as input and converts it into an image embedding that captures its visual representation. The second component is the prompt encoder, which enhances the SAM’s flexibility by supporting various types of prompts, including both sparse prompts (e.g., points, boxes, and text) and dense prompts (e.g., masks). Each of these prompt types utilizes distinct encoding methods. Dense mask prompts are initially embedded using convolutions and then combined with the processed image embedding through element-wise summation. Point and box prompts, on the other hand, are represented by positional encodings [42]. The final component is a mask decoder that integrates the image embedding and the output from the prompt encoder to generate the segmentation masks. Together, these interconnected components enable the SAM to create a highly adaptable segmentation model capable of delivering effective results.

**During testing,** the performance of the SAM was evaluated for the segmentation of road objects under various complex road conditions. Initially, an input image is processed by the SAM’s image encoder to generate an image embedding. The image encoder utilizes a ViT backbone, dividing the input image into non-overlapping patches that are then processed through a standard transformer encoder (MAE) to capture global contextual relationships. This study employed the SAM’s “Segment Everything” mode, which generates masks for all potential objects without any predefined regions or specific prompts. This approach is suited for road object segmentation due to the variable locations of road objects in different images, which makes fixed prior definitions impractical. Furthermore, no pre-processing, fine-tuning, or post-processing was applied to ensure an unbiased assessment of the SAM’s inherent segmentation capabilities. Unlike prompt-based evaluations that rely on prior object location knowledge, our study aimed to assess the SAM’s zero-shot segmentation ability on road scenes without such guidance, establishing a baseline understanding of its raw performance in this challenging context. The SAM output is a set of candidate object regions encompassing all potential objects in the image.

### 3.2. Datasets

The experiments in this study were conducted using three public datasets: KITTI [16], BDD100K [17], and Mapillary Vistas [18]. A comprehensive overview of the key characteristics of each selected dataset is provided in Table 2. Further details on each dataset are provided below.

**The KITTI dataset** (https://www.cvlibs.net/datasets/kitti/eval_instance_seg.php?benchmark=instanceSeg2015 accessed on 1 February 2025) consists of 400 images (200 for training and 200 for testing), each with a resolution of 1240 × 376 pixels. This dataset uses the same name and instance classes as the Cityscapes dataset.

**The BDD100K dataset** (https://www.kaggle.com/datasets/solesensei/solesensei_bdd100k accessed on 1 February 2025) is a large-scale vision dataset that contains 10,000 images (7000 for training, 1000 for validation, and 2000 for testing), each with a resolution of 1280 × 720, which are classified into 19 classes, providing the ground truth for semantic instance segmentation. These images were captured in various crowd scenes under various weather conditions (clear, snow, rain, fog, cloud, and partial cloud), as well as at different times of day (daytime, nighttime, dawn, or dusk). The purpose of capturing these variations is to reflect real-world scenarios that can occur during autonomous driving.

**The Mapillary Vistas dataset** (https://www.mapillary.com/dataset/vistas accessed on 1 February 2025) is considered one of the largest street-level datasets for image segmentation. It consists of 25,000 high-quality images divided into three categories: training (18,000 images), validation (2000 images), and testing (5000 images). The dataset contains 66 classes and has a resolution of 1920 × 1080. These images were captured in six different countries and depict various lighting conditions, seasons, weather scenarios, and times of day.

### 3.3. Experimental Settings

**Code Implementation:** The testing pipeline for the SAM in this study was primarily implemented following the guidelines provided in the official GitHub repository (https://github.com/facebookresearch/segment-anything accessed on 1 February 2025). The model provides three backbone variants of varying sizes for image encoding: ViT-H (Huge), ViT-L (Large), and ViT-B (Base). Our experiments evaluated all three backbones: ViT-H, comprising 32 transformer layers and 636M parameters; ViT-L, with 24 transformer layers and 308M parameters; and ViT-B, featuring 12 transformer layers and 91M parameters [9]. Furthermore, the “Segment Everything” mode was implemented using the SamAutomaticMaskGenerator class, an automated tool within the segment_anything library designed to generate predicted segmentation masks for all discernible objects in a given image.

**Package Versions:** All experiments were conducted using Python (version 3.10.12), PyTorch (version 2.5.1), and torchvision (version 0.20.1). OpenCV (version 4.10.0.84) and Matplotlib (version 3.10.0) were utilized for visualization purposes. The computational hardware consisted of an NVIDIA Tesla T4 GPU (15GB) with CUDA version 12.1.

### 3.4. Evaluation Metrics

In this experiment, we assessed the SAM’s performance using several common metrics for image segmentation tasks, as outlined below:

- **Intersection over Union (IoU):** IOU represents the ratio of the area of overlap ($|A_{i}\cap B_{i}|$) to the area of union ($|A_{i}\cup B_{i}|$) between the ground-truth mask $A_{i}$ and the predicted mask $B_{i}$ for each instance denoted by *i*, as expressed in Equation (1):
  (1) $${\mathrm{IoU}}_{i}=\frac{\left|A_{i}\cap B_{i}\right|}{\left|A_{i}\cup B_{i}\right|}$$
- **Mean Intersection over Union (mIoU):** mIoU represents the average IoU across all instances of a given class, denoted by *c*, as defined in Equation (2), where $M_{c}$ is the number of instances of class *c*, and ${\mathrm{IoU}}_{i,c}$ is the IoU of the *i*-th instance belonging to class *c*. In autonomous vehicle systems, mIoU reflects how accurately road objects are segmented. A higher mIoU indicates better scene understanding and contributes directly to safer navigation. In prior works [23,24,25,26,27,28,29], these methods have achieved mIoU scores ranging from 30.7% to 51.8%, which serves as a performance baseline. The SAM aims to approach or exceed this established range.
  (2) $${\mathrm{mIoU}}_{\mathrm{class}\;c}=\frac{1}{M_{c}}\sum_{i=1}^{M_{c}}{\mathrm{IoU}}_{i,c}$$
- **Highest IoU per Instance:** For each ground-truth instance $A_{i}$, the Highest IoU is computed as the maximum IoU with any predicted mask, as defined in Equation (3), where $B_{ij}$ is the *j*-th predicted mask compared to $A_{i}$, and $n_{i}$ is the number of predicted masks considered. This metric captures the model’s ability to accurately segment objects even when multiple or noisy predictions exist.
  (3) $${\mathrm{IoU}}_{i}^{\max}=\max_{j\in \{1,\ldots ,n_{i}\}}\frac{\left|A_{i}\cap B_{ij}\right|}{\left|A_{i}\cup B_{ij}\right|}$$
- **Average Highest IoU per Class:** This metric averages the Highest IoU values for a given class *c* across all images containing at least one instance of that class. It indicates how consistently the model performs across varied scenes for each class. It is defined in Equation (4). Where: ${\mathrm{IoU}}_{ck}^{\max}$ is the average Highest IoU for class *c* in image *k*, and $N_{c}$ is the number of images containing at least one instance of class *c*.
  (4) $${\mathrm{IoU}}_{\mathrm{class}\;c}^{\mathrm{avg}}=\frac{1}{N_{c}}\sum_{k=1}^{N_{c}}{\mathrm{IoU}}_{ck}^{\max}$$
- **Precision, Recall, and F1 Score:** These metrics are included to provide insight into the quality of the predicted masks and indicate whether the SAM successfully segments all relevant objects. This is particularly important in a zero-shot setting, where model uncertainty is higher due to the lack of task-specific training.
  **Precision** measures the ratio of correctly predicted positive instances to all predicted positives, as defined in Equation (5). High Precision reduces false positives, contributing to more reliable and trustworthy decision-making in autonomous systems. **Recall** measures the ratio of true positive predictions to all actual positive instances, as defined in Equation (6). In autonomous vehicle systems, high Recall ensures that critical road objects are not missed, which is vital for operational safety. **F1 Score** is the harmonic mean of Precision and Recall, as defined in Equation (7). Where: True Positive (TP) denotes the number of actual road objects that were correctly segmented, False Positive (FP) denotes the number of predicted road objects that were incorrectly identified as present, and False Negative (FN)denotes the number of actual road objects that were missed and not segmented.
  (5) $$\mathrm{Precision}=\frac{\mathrm{TP}}{\mathrm{TP}+\mathrm{FP}}$$
  (6) $$\mathrm{Recall}=\frac{\mathrm{TP}}{\mathrm{TP}+\mathrm{FN}}$$
  (7) $$F1\;\mathrm{Score}=2\cdot \frac{\mathrm{Precision}\cdot \mathrm{Recall}}{\mathrm{Precision}+\mathrm{Recall}}$$

## 4. Results and Discussion

In this section, both quantitative and qualitative results are presented to evaluate the SAM’s performance in road object segmentation. The analysis was aimed at revealing its strengths and identifying the challenges it faces in achieving accurate segmentation in complex road scenes, offering insights into its practical effectiveness and limitations.

### 4.1. Quantitative Results

This subsection presents a quantitative evaluation of the SAM for road object segmentation through several evaluations. All evaluations were conducted based on the SAM using different ViTs for image encoding in the “Segment Everything” mode to assess its performance under various road, weather, and illumination conditions. These results offer a comprehensive understanding of the SAM’s capabilities and challenges for segmenting road objects in diverse real-world scenarios.

#### 4.1.1. Evaluating SAM’s Segmentation Performance Under Different Vision Transformer Backbones

This experiment was performed to evaluate the segmentation performance of the SAM for road objects using three distinct vision transformer (ViT) backbones for image encoding: ViT-H (Huge), ViT-L (Large), and ViT-B (Base). The primary objective was to analyze the influence of the backbone size on the SAM’s ability to segment various road object categories. The evaluation was conducted on three public datasets (detailed in Section 3.2) utilizing the SAM’s “Segment Everything” mode. The quantitative results, focusing on the Mean Intersection over Union (mIoU) per class and the average of the Highest IoU achieved per class, are presented for the training sets in Table 3, Table 4 and Table 5 and for the validation sets in Table 6 and Table 7.

The tables presented above provide a comprehensive overview of class-specific segmentation performance, offering detailed insights into the SAM’s capabilities when using different backbones. The optimal ViT backbone varies across the datasets. As shown in Table 3, ViT-L achieves the highest Mean IoU on the KITTI dataset. For the BDD100K training set, as shown in Table 4, ViT-L and the smaller ViT-B exhibit comparable performance, slightly outperforming ViT-H. Similarly, in the validation set of the BDD100K dataset, as presented in Table 6, all three backbones produce similar mIoU results. Conversely, ViT-B demonstrates the best performance on both the training and validation sets, as presented in Table 5 and Table 7, respectively, of the Mapillary Vistas dataset. These performance variations are likely influenced by differences in annotation strategies. Specifically, KITTI and BDD100K employ a combination of instance and semantic segmentation masks. In these datasets, classes such as bicycles, buses, cars, caravans, motorcycles, persons, riders, trains, and trucks are annotated with instance masks, where each individual object has a separate mask. In contrast, the Mapillary Vistas dataset employs instance-level masks for all object categories.

**Figure 2:**
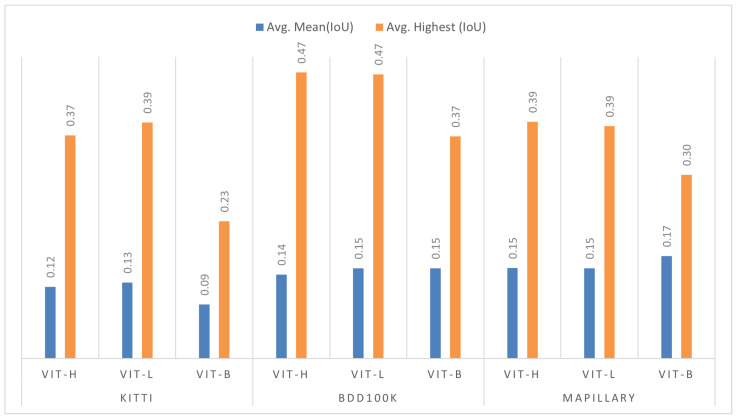
Average performance difference between Mean Intersection over Union (mIoU) and Highest Intersection over Union (Highest IoU) for ViT-H, ViT-L, and ViT-B on the KITTI, BDD100K, and Mapillary Vistas datasets.

Despite the relatively modest mIoU scores achieved in the “Segment Everything” mode, a clearer picture of the SAM’s potential emerges when examining the per-class average of the Highest IoU across datasets. As illustrated in Figure 2, these Highest IoU masks reflect a notably improved alignment with the ground truth, indicating the SAM’s ability to produce high-quality segmentations. This suggests that, although “Segment Everything” introduces noise and redundancy, the SAM can achieve substantially better performance through effective post-processing, such as filtering duplicate and low-quality masks.

Consequently, our evaluation of the SAM with different ViT backbone sizes in a zero-shot setup reveals a clear trend: while increasing model size can enhance segmentation quality, the performance gains diminish beyond a certain point. Specifically, ViT-L (24 layers, 308M parameters) and ViT-H (32 layers, 636M parameters) yield nearly identical results across all datasets in both average Mean IoU and Highest IoU, as shown in Figure 2. This observed similarity, despite ViT-H’s significantly greater depth and parameter count, is attributable to the inherent limitations of a zero-shot configuration. In such general-purpose setups, the model’s increased capacity does not translate into proportional performance improvements, likely due to the absence of domain-specific fine-tuning or prompt adaptation that would enable the larger model to effectively exploit its additional parameters and deeper architecture. ViT-L’s architecture appears to offer an optimal trade-off: it consistently delivers high segmentation quality comparable to ViT-H, but with significantly reduced computational demands. While ViT-B slightly outperforms ViT-H and ViT-L in terms of average Mean IoU on the Mapillary dataset, this result is not consistent across datasets, and its Highest IoU remains noticeably lower. This suggests that the smaller architecture may occasionally benefit from better generalization on specific datasets but lacks the broader segmentation accuracy and robustness of larger models.

#### 4.1.2. Evaluating SAM’s Segmentation Performance Without Explicit Prompting

**Figure 3:**
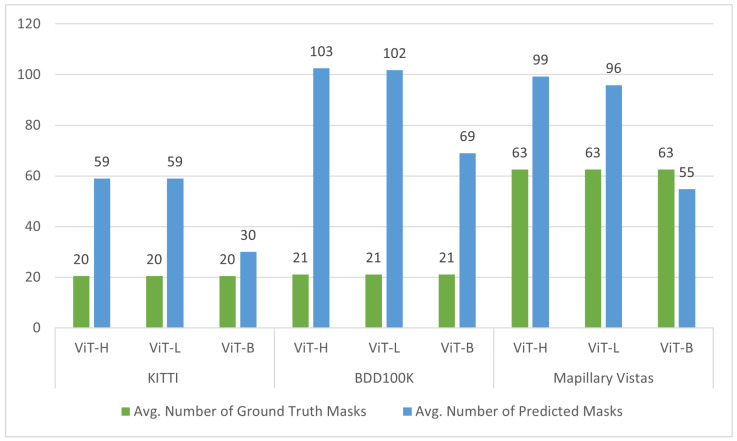
A comparison of the average number of ground-truth masks and the average number of predicted masks generated by the SAM using different ViT backbones on the KITTI, BDD100K, and Mapillary Vistas datasets.

This study evaluated the SAM using the “Segment Everything” mode, which operates without explicit prompts. The results reveal significant over-segmentation across all vision transformer backbones (ViT-H, ViT-L, and ViT-B). In particular, ViT-H and ViT-L tend to cause more over-segmentation than ViT-B, as evidenced by the considerably higher number of predicted masks compared to ground-truth masks, as depicted in Figure 3. For instance, for the KITTI dataset, ViT-H and ViT-L predict approximately three times the number of ground-truth masks, while ViT-B predicts around one and a half times more. Similarly, with the BDD100K dataset, ViT-H and ViT-L exhibit a higher degree of over-segmentation, predicting five times the number of ground-truth masks, compared to ViT-B’s three and a half times. This trend continues in the Mapillary Vistas dataset, with ViT-H and ViT-L showing a substantial increase in predicted masks. Interestingly, ViT-B demonstrates slight under-segmentation with this dataset, as the number of predicted masks is smaller than the number of ground-truth masks.

**Figure 4:**
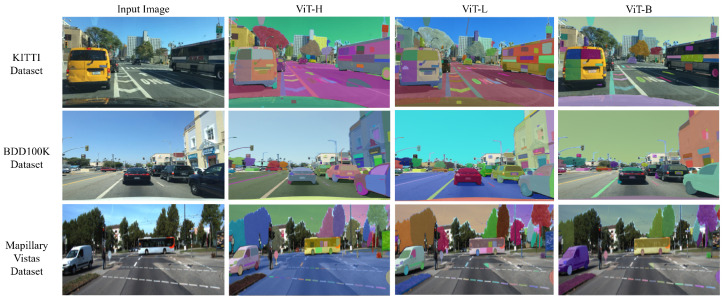
Visual analysis of the SAM’s segmentation performance without explicit prompting using different ViT backbones on the KITTI, BDD100K, and Mapillary Vistas datasets.

This observation of over-segmentation is potentially related to the complexity of feature representations learned by different ViT architectures. Higher-capacity ViT models, such as ViT-H (32 transformer layers, 636M parameters) and ViT-L (24 transformer layers, 308M parameters), with their inherently greater depth and increased number of parameters, significantly enhance their ability to capture more details. This increased capacity can lead the SAM to misinterpret distinct parts of a single road object instance as separate entities when operating in its “Segment Everything” mode. This behavior arose because, in this study, the SAM was evaluated without explicit prompts, and thus, lacking a specific focus on particular objects, it attempted to segment everything it perceived as a distinct visual element. Consequently, the rich feature representations of larger ViTs can inadvertently contribute to this fragmentation of coherent road objects. This phenomenon is illustrated in Figure 4, where each of the three images from different datasets showcases the over-segmentation behavior in the absence of object-specific prompting. Objects such as cars and buses are segmented into multiple masks, with individual components such as wheels and windows being delineated as separate objects. Similarly, road lane markings are often fragmented into multiple masks, with each lane segment treated as a distinct predicted mask. ViT-L demonstrates comparable over-segmentation behavior, with both ViT-H and ViT-L generating a similar number of predicted masks, as detailed in Figure 3. Conversely, ViT-B, with its reduced model capacity, exhibits a lower propensity for over-segmentation. As shown in Figure 4, ViT-B results in fewer instances of object splitting. For example, the number of predicted masks for objects such as buses and cars is lower than the masks generated by ViT-H and ViT-L, as ViT-B does not capture the same level of detail. However, ViT-B also illustrates challenges in detecting and segmenting some road objects. Figure 4 demonstrates that the ViT-B fails to completely or partially segment some road objects, such as road areas or parts of car objects. This suggests that while ViT-B mitigates over-segmentation, it may struggle to capture the full extent of certain objects, potentially leading to under-segmentation. These observations regarding over-segmentation help explain the low Mean IoU scores obtained with all datasets and different ViT backbones, as presented in Table 3, Table 4 and Table 5.

#### 4.1.3. Evaluating SAM’s Inference Time with Different ViT Backbones

Inference time is a critical factor in evaluating model performance, particularly for real-time applications. Table 8 presents the average inference time (in seconds) for processing 100 randomly selected images and the corresponding mIoU results for different ViT backbones across three datasets: KITTI, BDD100K, and Mapillary Vistas. The results demonstrate a consistent trade-off across all datasets; smaller backbones (ViT-B) exhibit faster inference times but tend to yield lower mIoU values. However, it is crucial to contextualize these findings within the stringent real-time requirements of autonomous driving systems, which typically demand processing speeds of 30 frames per second (fps) or more (i.e., approximately 33 milliseconds per frame). Our observed average inference times, ranging from 4.80 to 8.45 s per image, translate to an approximate processing speed of between 0.12 and 0.21 fps. This clearly indicates that the current zero-shot SAM implementation, in its unoptimized form, does not meet real-time requirements for autonomous driving applications. Specifically, ViT-B achieved inference times of 4.80 s, 5.30 s, and 5.72 s on the KITTI, BDD100K, and Mapillary Vistas datasets, respectively, while the corresponding mIoU results were 0.08, 0.12, and 0.12. This lower mIoU is likely attributable to the reduced number of masks predicted by ViT-B (30, 50, and 51 masks, respectively), indicating potential object under-segmentation. Conversely, larger models (ViT-H and ViT-L) possess the capacity to capture more complex patterns, which leads to a greater computational demand.

In this experiment, ViT-L offered a more favorable balance between processing speed and segmentation accuracy (mIoU). Specifically, on the KITTI dataset, although the average number of predicted masks (59) was comparable to that predicted by ViT-H (59), ViT-L achieved a significantly lower inference time of 5.61 s with the same mIoU of 0.10. A similar trend was observed on BDD100K, where ViT-L’s inference time (6.95 s) was notably better than ViT-H’s (8.45 s) while achieving a superior mIoU of 0.13 with an average of 87 predicted masks compared to 88 for ViT-H. On the Mapillary Vistas dataset, ViT-L again demonstrated a faster inference time (6.47 s) and a higher mIoU (0.15) compared to ViT-H (7.68 s and 0.14, respectively), with an average of 91 predicted masks compared to 94 for ViT-H. These results strongly suggest that ViT-L presents a more efficient architecture for relative real-time applications, offering a better trade-off between speed and accuracy without a substantial increase in potential over-segmentation compared to the more computationally demanding ViT-H.

#### 4.1.4. Evaluating SAM’s Segmentation Performance with Different Road Object Sizes

The size of road objects within images varies significantly due to their actual physical dimensions and their distance from the camera sensor. Distant objects occupy fewer pixels compared to those located closer to the sensor. To further evaluate the segmentation performance of the SAM in road object segmentation, we analyzed its efficacy using different object sizes within the image. Utilizing a percentile-based categorization, road objects were classified into three categories: small, medium, and large. Objects with a pixel count below the 25th percentile were designated as small, those above the 75th percentile as large, and those falling between these thresholds as medium. This classification can be summarized as follows:

$$\mathrm{Object}\;\mathrm{Size}=\left\{\begin{array}{cc} \mathrm{Small} & \mathrm{if}\;\mathrm{pixel}\;\mathrm{count}<25\mathrm{th}\;\mathrm{percentile}\\ \mathrm{Medium} & \mathrm{if}\;25\mathrm{th}\;\mathrm{percentile}\leq \mathrm{pixel}\;\mathrm{count}\leq 75\mathrm{th}\;\mathrm{percentile}\\ \mathrm{Large} & \mathrm{if}\;\mathrm{pixel}\;\mathrm{count}>75\mathrm{th}\;\mathrm{percentile} \end{array}\right.$$

Our analysis revealed that the SAM’s performance generally follows a trend of better segmentation for larger objects, followed by medium-sized objects, with the most challenges occurring for small objects across all datasets and ViT backbones. Specifically, ViT-H and ViT-L showed closely aligned performance and consistently outperformed ViT-B. This trend is evident in Table 9, which presents the average IoU results for each object size category on the KITTI, BDD100K, and Mapillary Vistas datasets. For instance, on the KITTI dataset, ViT-H achieved an average IoU of 0.64 for large objects, 0.48 for medium objects, and 0.12 for small objects. This pattern was consistently observed across all datasets and backbones, reinforcing the observation that the SAM’s segmentation accuracy is positively correlated with object size.

**Figure 5:**
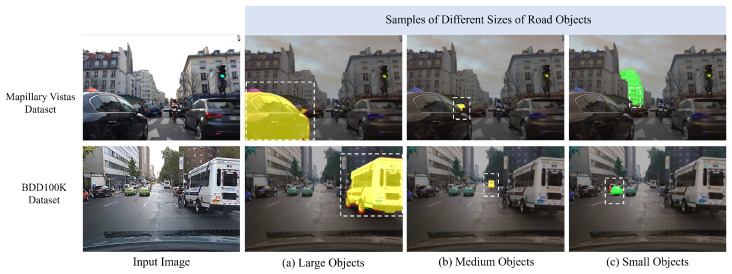
The SAM’s segmentation performance with different road object sizes. Yellow masks represent the overlap between the ground-truth mask (red) and the predicted masks generated by the SAM (green). White dashed rectangles indicate road objects of varying sizes selected for analysis.

**Figure 6:**
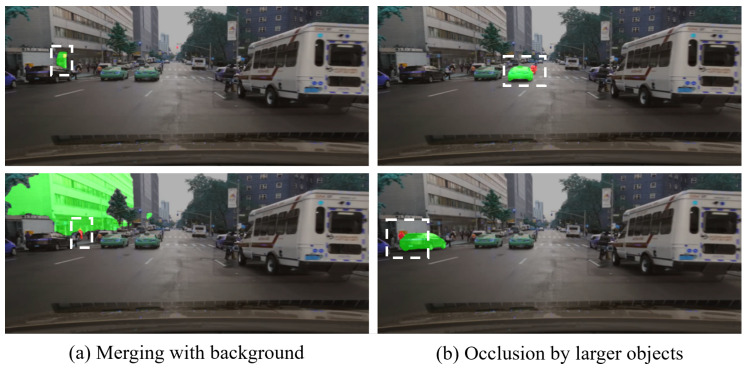
The SAM’s performance in small road object segmentation. Ground-truth masks are in red, and the SAM’s predicted masks are in green, with yellow masks showing the overlapped area (BDD100K dataset sample). White dashed rectangles indicate specific small road objects selected for analysis.

Visual evidence is presented to further validate the numerical findings, as shown in Figure 5. The analysis demonstrated the SAM’s proficiency in accurately segmenting large objects with clear boundaries, as well as medium-sized objects, as shown in Figure 5a,b. However, the SAM encounters challenges when segmenting small road objects, which are often influenced by surrounding contextual factors, as illustrated in Figure 5c. To further illustrate the challenges faced by the SAM in segmenting small objects, Figure 6 shows various scenarios where these difficulties arise. Specifically, small objects are frequently merged with larger background elements, as illustrated in Figure 6a, where small objects, such as a traffic sign or a person, are partially or fully merged with large objects, such as a building in the background. This merging phenomenon obscures the boundaries of the small objects, leading to inaccurate segmentation. Furthermore, occlusion or overlap with larger objects poses another significant challenge. As shown in Figure 6b, the motorcycle and a person are partially occluded by a larger object, namely, a car. This occlusion hinders accurate segmentation, as the boundaries of small objects become obscured and indistinguishable from the occluding object. These examples highlight the vulnerability of small object segmentation to contextual factors such as background interference and occlusion, which contribute to the inaccurate segmentation observed in the quantitative results.

#### 4.1.5. Detailed Performance Analysis

The previous metrics primarily evaluate the SAM based on what it localizes and segments, but they do not provide insight into the quality of the predicted masks or whether the SAM successfully segments all objects. In this subsection, we further evaluate the SAM’s performance using additional metrics, including Recall, Precision, and F1 Score, as shown in Table 10, Table 11 and Table 12 for the KITTI, BDD100K, and Mapillary Vistas datasets, respectively. The experiments were conducted using the Highest IoU for each instance to calculate these metrics, and we employed different thresholds (i.e., 0.2, 0.5, and 0.7) to assess the performance of the SAM’s predicted masks. For all datasets and backbones, lowering the threshold generally increases Recall, indicating that the model can capture more road objects. The F1 Score tends to peak at a lower threshold (0.2). In terms of backbones, ViT-H and ViT-L show very similar performance, with slight variations depending on the dataset, as they consistently outperform ViT-B in terms of F1 Score, Precision, and Recall.

To assess the road object segmentation capabilities of the different ViT backbones, we analyzed False Negative (FN) counts using the three datasets. As detailed in Table 10, on the KITTI dataset (comprising 4087 objects), ViT-H missed 851 road object instances, ViT-L missed 916 instances, and ViT-B missed 1415 instances. This corresponds to missed detection and segmentation rates of approximately 21% for ViT-H, 22% for ViT-L, and 35% for ViT-B. Moving to the BDD100K dataset, as shown in Table 11 (comprising 147,819 objects), ViT-H and ViT-L demonstrated nearly identical performance in terms of FN, failing to segment 20,653 and 20,632 instances, respectively. In contrast, ViT-B missed 36,866 instances. These figures translate to missed segmentation rates of 14% for ViT-H and ViT-L and 25% for ViT-B. Furthermore, for the Mapillary Vistas dataset, the results of which are presented in Table 12 (comprising 1,125,932 road objects), the number of missed road object segmentations was 381,513 for ViT-H, 407,261 for ViT-L, and 598,865 for ViT-B. This resulted in missed segmentation rates of 34% for ViT-H, 36% for ViT-L, and 53% for ViT-B. In particular, the FN rate for Mapillary Vistas is significantly higher than that for the KITTI and BDD100K datasets. This substantial increase in the FN rate is attributed to the Mapillary Vistas dataset’s inherent complexity, characterized by diverse scenes, varying lighting conditions, and a higher density of small objects, which pose significant challenges for accurate segmentation.

These findings consistently demonstrate that ViT-H and ViT-L significantly improve the F1 Score and reduce FN rates compared to ViT-B across all datasets. This highlights the importance of employing larger model capacities for accurate road object segmentation, particularly in autonomous vehicle applications, where comprehensive and precise segmentation of road objects is crucial for ensuring safety.

### 4.2. Qualitative Analysis

The qualitative analysis aims to provide a deeper understanding of the SAM’s segmentation performance beyond quantitative metrics. Although quantitative findings offer insight into overall accuracy and efficiency, they do not always capture the specific challenges that arise in complex road scenes. By visually inspecting the SAM’s outputs, we highlight the challenges that hinder its ability to accurately segment road objects. Before analyzing specific qualitative challenges, it is important to consider potential underlying causes. One key factor is the nature of the SAM’s training dataset, SA-1B, which predominantly consists of natural images and lacks detailed representations of structured road scenes. As a result, critical features such as lane markings, reflective surfaces, and complex traffic environments are underrepresented. This domain gap may contribute to the SAM’s difficulty in segmenting certain road-specific elements, as discussed in the following subsections.

#### 4.2.1. Visual SAM Segmentation Performance with Ambiguous Borders Between Road Objects

**Figure 7:**
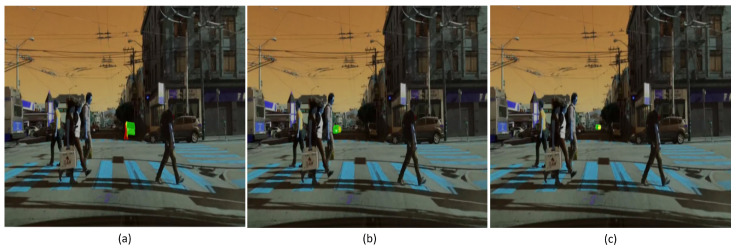
A border ambiguity challenge, where the SAM’s predicted masks (green) struggle to accurately delineate the boundaries of adjacent objects, as in (a), and overlapping road objects, as in (b) and (c). Ground-truth masks are shown in red, and yellow masks represent the overlap between the prediction and the ground truth (Mapillary Vistas dataset sample).

A visual analysis of the SAM’s performance in road object segmentation revealed a significant challenge stemming from border ambiguity, characterized by the SAM’s difficulty in accurately delineating object boundaries, particularly when road objects are closely positioned or overlapping. As illustrated in Figure 7, the SAM exhibits these limitations in several instances. In Figure 7a, the SAM incorrectly segments a person by merging the person’s mask with that of an adjacent car. The yellow mask clearly highlights the area where the SAM struggles to correctly define the border between these two distinct objects, leading to the car being erroneously considered part of the person. Furthermore, Figure 7b,c demonstrate the SAM’s difficulty in accurately segmenting individual cars when they are adjacent. Instead of generating separate masks for each vehicle, the SAM incorrectly merges the adjacent cars into a single, larger predicted mask, treating them as a single object. These visual examples underscore the challenge that border ambiguity poses for the SAM in accurately distinguishing and segmenting closely situated or overlapping road objects. This challenge of border ambiguity, leading to merging errors, negatively impacts segmentation performance, resulting in an increase in false positives and a subsequent decrease in the Mean IoU for specific object instances.

#### 4.2.2. Visual SAM Segmentation Performance for Road Objects with Various Colors and Textures

**Figure 8:**
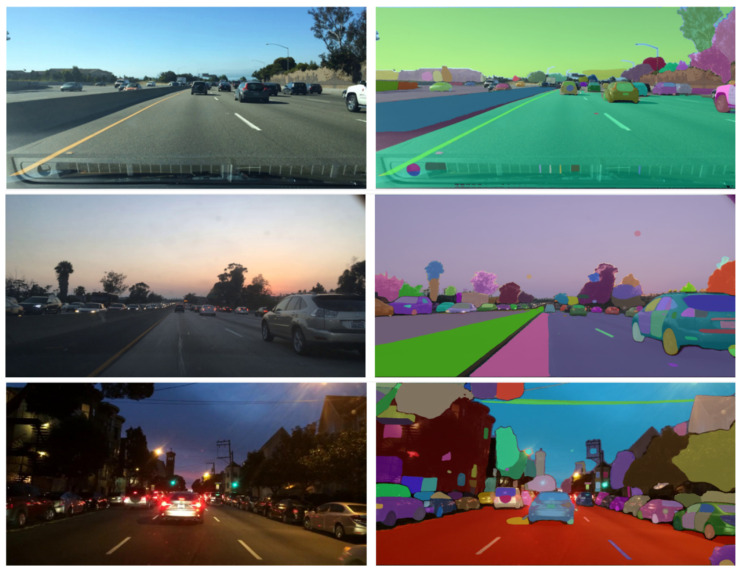
The impact of color and texture variations on the SAM’s segmentation. Sample images from the BDD100K and Mapillary Vistas datasets.

The inherent diversity of road objects often presents significant variations in color and texture, which poses a notable challenge for segmentation models. This limitation is clearly illustrated in Figure 8. Specifically, the first image demonstrates the SAM’s tendency to over-segment the road surface, likely resulting from variations in asphalt conditions. The second image further underscores this issue, showing how even a partially snow-covered road can cause the SAM to fragment the road area into multiple distinct masks. This over-segmentation behavior is not limited to the road surface; other crucial road objects also exhibit this characteristic. For example, vehicles or buildings that have variations in color or materials, as illustrated in the third image of Figure 8, can also be over-segmented. Consequently, this inherent sensitivity to intra-object variations significantly hinders the SAM’s ability to produce consistent segmentations, leading to over-segmentation and potentially impeding accurate object segmentation in complex road scenes.

#### 4.2.3. Challenges Related to Road-Specific Environmental Factors: Shadow- and Reflection-Induced Mis-Segmentation

Shadows and reflections are common in road scenes and pose significant challenges for efficient road object segmentation. This section visually depicts and analyzes the effect of shadows and reflections on the SAM’s segmentation performance.

**Figure 9:**
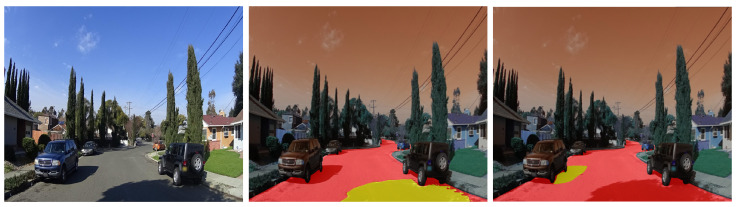
The SAM’s incorrect segmentation of shadows as separate objects. The yellow mask shows the overlap between the ground-truth mask (red) and the SAM’s predicted mask (green) (Mapillary Vistas dataset sample).

**Figure 10:**
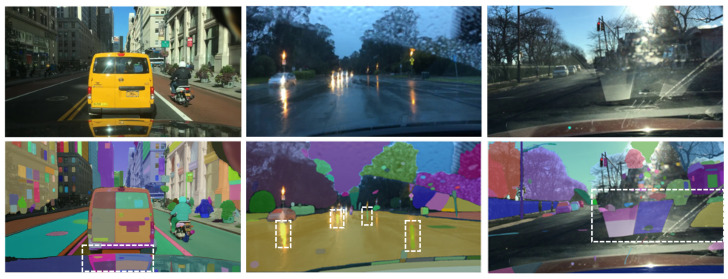
The SAM’s incorrect segmentation of reflections. White dashed rectangles in the predicted masks highlight areas where reflections were erroneously segmented as distinct objects (BDD100K dataset samples).

SAM frequently treats shadows as separate objects, resulting in incorrectly predicted masks. As depicted in Figure 9, the SAM generated masks for the shadows of cars and trees on the road, demonstrating its difficulty in distinguishing actual objects from their shadows. Furthermore, the SAM exhibits challenges in handling reflections. It mistakenly segments reflections on car bodies, wet surfaces, or reflections of the car dashboard on the front windshield as actual objects, as presented in Figure 10. These mis-segmentations contribute to an increase in false positives, negatively impacting the segmentation results, particularly for objects overlapping with shadows or reflections. The incorrectly predicted masks are often incorporated as part of the overlapped object instance, leading to a decrease in the Mean IoU. For instance, in Figure 9, the shadow cast by the car or tree is segmented as a separate mask. When this shadow overlaps with the road surface, it is considered part of the road object during the evaluation. This inclusion of the shadow mask within the road mask leads to a lower IoU for the road object. Consequently, the overall Mean IoU for road objects is negatively impacted due to the inclusion of these erroneous shadow masks. These findings underscore the sensitivity of the SAM to road-specific environmental factors such as shadows and reflections that pose a notable obstacle to achieving accurate and reliable road object segmentation.

#### 4.2.4. Challenges Related to Weather Conditions

**Figure 11:**
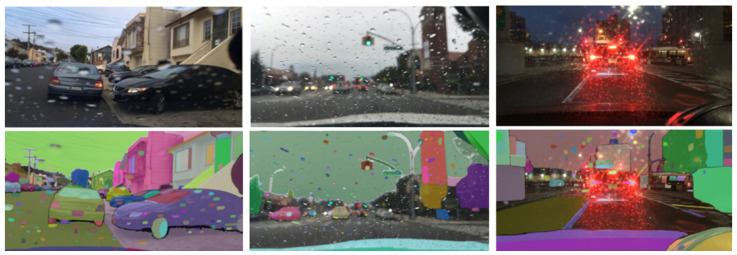
SAM’s segmentation performance in rainy weather (BDD100K dataset samples).

Road scenes are inherently subject to diverse weather conditions that can significantly alter visual appearance, consequently affecting the performance of image segmentation models. Rainy conditions, a prevalent weather-related factor in road environments, present a notable challenge to the SAM’s segmentation capabilities. As illustrated in Figure 11, the SAM demonstrates a tendency to misinterpret individual raindrops on the windshield as discrete entities, generating spurious masks for each instance. This misinterpretation adversely affects the segmentation of objects occluded by or overlapping with these raindrops, resulting in inaccurate object boundaries and a reduction in the mean (IoU) due to the erroneous inclusion of raindrop masks. Furthermore, this phenomenon can lead to over-segmentation, wherein single road objects are fragmented into multiple masks, including those erroneously attributed to raindrops, thereby impeding precise object identification and delineation in rainy scenes.

#### 4.2.5. Challenges Related to Illumination Conditions

**Figure 12:**
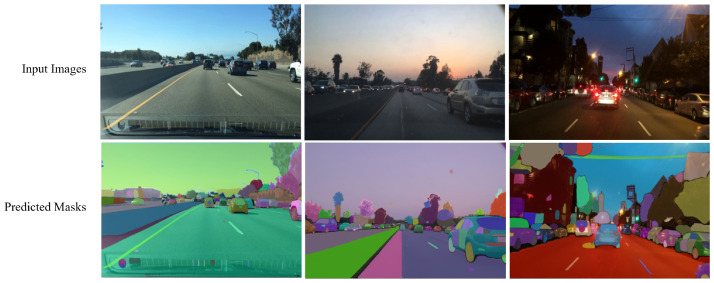
The SAM’s segmentation performance at different times of day: daytime and twilight (BDD100K dataset samples).

**Figure 13:**
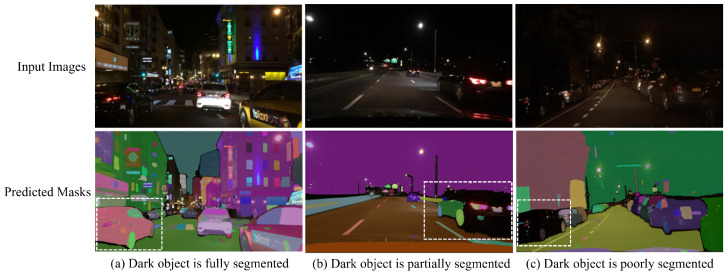
The SAM’s segmentation performance at night or under low illumination. White dashed rectangles indicate dark road objects selected for analysis in nighttime scenes. (BDD100K dataset samples).

The SAM’s performance was evaluated under varying illumination conditions: daytime, twilight, and nighttime. As demonstrated in Figure 12, the SAM generally achieved a robust segmentation of road objects exhibiting clear boundaries during daytime and twilight. In contrast, nighttime conditions presented significant challenges to its performance. However, as shown in Figure 13a, the SAM successfully segmented road objects at night when they were illuminated by lights and sufficiently contrasted with the background. For instance, the black car highlighted by a white dashed rectangle in Figure 13a was detected and segmented due to this contrast and its clear border. In contrast, the SAM struggled to segment or detect dark-colored road objects against dark backgrounds, as illustrated in Figure 13b,c. Specifically, in Figure 13b, the black car is only partially segmented, with the SAM being unable to distinguish the remainder of the car body from the dark sky. Furthermore, in Figure 13c, the dark-colored car is not segmented, with the SAM generating masks only for the wheels. Consequently, the consistency of the SAM’s segmentation accuracy for dark objects at night is compromised when compounded by challenging road conditions such as low illumination and unclear object borders resulting from minimal contrast against a dark background.

## 5. Challenges and Recommendations

Our comprehensive evaluation of the SAM for zero-shot road object segmentation, conducted without explicit prompts, revealed several key challenges that hinder its accurate and robust performance. Firstly, we observed a crucial trade-off between segmentation accuracy and computational efficiency across different ViT backbones (ViT-H, ViT-L, ViT-B). While larger backbones (ViT-H and ViT-L) generally yielded superior segmentation quality, their increased processing demands pose challenges for real-time autonomous driving systems, where low latency and precise perception are critical for safety, contrasting with the faster inference but reduced accuracy of ViT-B. Secondly, the inherent use of the “Segment Everything” mode resulted in significant over-segmentation, leading to a relatively low overall Mean IoU across all backbones. This issue was often exacerbated by the finer details captured by larger ViTs, which generated more masks. Furthermore, the SAM’s segmentation performance significantly correlated with object size, demonstrating greater segmentation proficiency with larger objects while struggling with smaller ones, particularly those affected by occlusion and background clutter. Finally, the model exhibited significant sensitivity to prevalent environmental factors in road scenes, including shadows, reflections (which it often failed to differentiate from actual objects), and adverse weather and illumination conditions, where rain introduced visual noise, further hindering performance. Moreover, the SAM struggled with border ambiguity, failing to accurately delineate object boundaries for closely positioned or overlapping road objects.

Building upon the insights gained from this evaluation of the SAM’s performance for road object segmentation, future research should explore strategies to overcome the identified challenges and enhance its applicability in real-world road scenarios. We propose the following key recommendations: Firstly, a key direction for future research involves significantly enhancing the SAM’s segmentation performance and robustness in real-world autonomous driving scenarios. This can be approached in two ways: (1) improving the model’s robustness to challenging road scene conditions, such as adverse weather, lighting variations, ambiguous object boundaries, and varying object sizes, through domain adaptation strategies such as fine-tuning on diverse road scene datasets or auto-prompt engineering. Since other studies have enhanced the SAM’s performance through fine-tuning and auto-prompt engineering in other domains, such as medical and remote sensing imagery, these techniques hold great potential for improving its robustness and accuracy in autonomous driving scenarios; (2) developing efficient post-processing techniques to address current limitations such as over-segmentation issues, pushing performance beyond established baselines (30.7–51.8%) [23,24,25,26,27,28,29]. Such a comprehensive approach will significantly improve the SAM’s flexibility and applicability across varied road environments. Secondly, addressing the significant computational demands of the SAM is paramount for its real-time applicability in autonomous systems. Future work should focus on exploring model optimization techniques, the development of lightweight SAM architectures, and hardware acceleration, which will be critical to achieving the high frame rates required for real-time autonomous perception. Thirdly, targeted investigations are needed to improve SAM’s performance on small and complex road objects, potentially through architectural modifications or specialized training strategies. Finally, to comprehensively benchmark the SAM’s practical utility, future studies should conduct quantitative performance comparisons with established state-of-the-art supervised segmentation models. Such comparisons, particularly after the domain-specific fine-tuning or sophisticated prompt engineering of the SAM, are essential for understanding its competitive standing relative to models specifically optimized for road object segmentation. These recommended research directions will be fundamental to enhancing the SAM’s foundational capabilities, transforming it into a robust and reliable solution for real-time road scene understanding in autonomous vehicles.

## 6. Conclusions

In this study, we comprehensively evaluated the Segment Anything Model (SAM) for the segmentation of road objects. Based on the results of tests conducted to evaluate the SAM’s performance in road object segmentation, our findings can be summarized as follows: (1) The SAM exhibits promising segmentation capabilities for certain road objects but struggles in challenging scenarios, particularly with small objects and ambiguous boundaries, which lead to unstable or inaccurate predictions. (2) Among the evaluated model variants, the SAM with a larger ViT backbone (ViT-H or ViT-L) generally produced higher segmentation quality than the smaller ViT-B model. ViT-L, in particular, offers an optimal balance between accuracy and inference time. However, the SAM’s current performance, even with ViT-L, falls short of the segmentation quality required for robust autonomous perception. Moreover, ViT-L’s inference speed remains far below the real-time requirements necessary for autonomous driving systems, which limits its applicability in real-world autonomous systems. (3) The SAM’s segmentation performance correlated with different factors inherent to road scenes, including object size, boundary complexity, and environmental conditions (e.g., shadows, reflections, and adverse weather). Overall, while the SAM shows potential as a general-purpose segmentation model, it is not yet sufficiently robust for deployment in autonomous vehicle applications. We hope that this study provides valuable insights into the SAM’s strengths and limitations in complex road environments, and we encourage future research to address these gaps, particularly through task-specific adaptation and computational optimization.

## Figures and Tables

**Table 1:** Comparison of semantic segmentation methods on multiple datasets.

Method	Dataset	Set	mIoU (%)
Enhanced FPN [23]	Mapillary Vistas	Validation set	43.6
MPNet [24]	Mapillary Vistas	Validation set	43.9
RGPNet [25]	Mapillary Vistas	Validation set	41.7
AGLNet [28]	Mapillary Vistas	Validation set	30.7
SFRSeg [26]	KITTI	Test set	49.3
SDBNet [27]	KITTI	Test set	51.8

**Table 2:** Comparison of datasets used for evaluation of SAM for road object segmentation.

Feature	KITTI	BDD100K	Mapillary Vistas
Primary Focus	Standard urban and rural driving scenarios	Diverse scenes across environmental conditions	Urban street-level scenes
Number of Classes	30	19	66
Weather Conditions	Clear weather	Clear, snow, rain, fog, cloud, partial cloud	Sunny, rainy, foggy, snowy
Time of Day Coverage	Daytime	Day, night, dawn, dusk	Day, night, twilight
Geographic Diversity	Low (Germany)	Moderate (different regions in USA)	High (different countries globally)

**Table 3:** A comparison of the SAM’s performance on the KITTI dataset using ViT-H, ViT-L, and ViT-B backbones, evaluated by Mean Intersection over Union (Mean IoU) and Highest Intersection over Union (Highest IoU) for each class. The best performance for each class and metric is highlighted in bold.

	ViT-H		ViT-L		ViT-B	
Class Name	Mean (IoU)	Highest (IoU)	Mean (IoU)	Highest (IoU)	Mean (IoU)	Highest (IoU)
Bicycle	0.14	**0.28**	**0.16**	**0.28**	0.14	0.21
Bridge	**0.06**	**0.15**	**0.06**	**0.15**	0.02	0.02
Building	**0.04**	**0.36**	**0.04**	0.34	0.03	0.21
Bus	**0.30**	**0.53**	0.24	0.45	0.25	0.36
Car	**0.30**	**0.72**	**0.30**	0.71	**0.30**	0.61
Caravan	0.34	0.68	**0.46**	**0.84**	0.05	0.10
Dynamic	0.03	0.09	**0.05**	**0.12**	0.01	0.04
Fence	0.07	**0.23**	**0.08**	0.21	0.04	0.11
Ground	**0.04**	0.11	**0.04**	**0.13**	0.03	0.07
Guard-rail	**0.08**	**0.37**	0.07	0.31	0.02	0.08
Motorcycle	**0.17**	**0.43**	0.15	0.39	0.01	0.01
Parking	**0.07**	**0.26**	**0.07**	0.24	0.05	0.13
Person	0.07	0.13	**0.08**	**0.15**	0.06	0.07
Pole	**0.03**	0.16	**0.03**	**0.17**	**0.03**	0.13
Polegroup	**0.01**	**0.01**	**0.01**	**0.01**	**0.01**	**0.01**
Rail_track	**0.13**	**0.42**	0.12	**0.42**	0.07	0.18
Rider	**0.13**	0.26	0.11	**0.41**	0.02	0.11
Road	**0.10**	**0.76**	0.09	0.71	0.06	0.26
Sidewalk	0.09	**0.35**	**0.11**	**0.35**	0.08	0.19
Sky	0.20	**0.90**	0.21	**0.90**	**0.30**	0.78
Static	0.07	**0.29**	**0.08**	**0.29**	**0.08**	0.24
Terrain	**0.08**	**0.43**	**0.08**	0.42	**0.08**	0.25
Traffic Light	**0.04**	**0.15**	**0.04**	0.12	**0.04**	0.10
Traffic Sign	0.19	**0.44**	0.18	0.42	**0.22**	0.40
Trailer	0.09	0.40	**0.12**	**0.50**	0.06	0.38
Train	**0.18**	**0.44**	0.12	0.42	0.09	0.34
Truck	0.23	0.44	**0.27**	**0.54**	0.23	0.40
Tunnel	0.15	0.51	**0.23**	**0.92**	0.11	0.43
Vegetation	0.06	**0.40**	0.06	0.39	**0.07**	0.33
Wall	0.10	0.39	**0.12**	**0.41**	0.11	0.30
**Average**	0.12	0.37	**0.13**	**0.39**	0.09	0.23

**Table 4:** A comparison of the SAM’s performance on the training set of the BDD100K dataset (19 classes) using ViT-H, ViT-L, and ViT-B backbones, evaluated by Mean Intersection over Union (Mean IoU) and Highest Intersection over Union (Highest IoU) for each class. The best performance for each class and metric is highlighted in bold.

	ViT-H		ViT-L		ViT-B	
Class Name	Mean (IoU)	Highest (IoU)	Mean (IoU)	Highest (IoU)	Mean (IoU)	Highest (IoU)
Bicycle	**0.18**	0.38	**0.18**	**0.39**	**0.18**	0.31
Building	**0.04**	**0.39**	**0.04**	0.37	**0.04**	0.24
Bus	**0.23**	**0.71**	**0.23**	0.70	0.20	0.53
Car	0.25	**0.63**	0.25	0.62	**0.26**	0.54
Fence	**0.10**	**0.41**	**0.10**	0.39	0.08	0.27
Motorcycle	0.20	**0.43**	**0.21**	0.42	0.18	0.32
Person	0.21	**0.40**	0.21	**0.40**	**0.23**	0.35
Pole	**0.06**	**0.25**	**0.06**	**0.25**	0.05	0.18
Rider	0.16	**0.35**	**0.17**	0.33	**0.17**	0.28
Road	**0.06**	**0.71**	0.05	0.62	0.04	0.32
Sidewalk	**0.07**	**0.38**	**0.07**	0.36	0.06	0.23
Sky	0.12	**0.84**	0.12	**0.84**	**0.16**	0.74
Terrain	**0.09**	**0.37**	**0.09**	0.36	0.08	0.26
Traffic Light	0.13	0.30	0.13	**0.31**	**0.16**	0.29
Traffic Sign	0.21	0.46	0.21	**0.47**	**0.26**	0.46
Train	0.20	**0.55**	**0.21**	**0.55**	0.17	0.46
Truck	**0.25**	0.47	**0.25**	**0.68**	**0.25**	0.54
Vegetation	**0.05**	**0.38**	**0.05**	0.37	**0.05**	0.28
Wall	**0.14**	**0.53**	**0.14**	0.52	**0.14**	0.40
**Average**	0.14	**0.47**	**0.15**	**0.47**	**0.15**	0.37

**Table 5:** A comparison of the SAM’s performance on the training set of the Mapillary Vistas dataset (66 classes) using ViT-H, ViT-L, and ViT-B backbones, evaluated by Mean Intersection over Union (Mean IoU) and Highest Intersection over Union (Highest IoU) for each class. The best performance for each class and metric is highlighted in bold.

	ViT-H		ViT-L		ViT-B	
Class Name	Mean (IoU)	Highest (IoU)	Mean (IoU)	Highest (IoU)	Mean (IoU)	Highest (IoU)
banner	0.29	0.51	0.30	0.50	**0.40**	**0.53**
barrier	**0.13**	**0.46**	0.12	0.45	**0.13**	0.37
bench	**0.19**	**0.35**	**0.19**	0.33	0.16	0.25
bicycle	0.14	**0.29**	**0.15**	**0.29**	0.13	0.19
bicyclist	**0.18**	**0.34**	0.17	0.32	**0.18**	0.26
bike-lane	**0.08**	**0.33**	0.07	0.32	0.07	0.22
bike-rack	**0.13**	**0.22**	**0.13**	**0.22**	0.10	0.14
billboard	0.29	0.50	0.30	0.51	**0.40**	**0.53**
bird	0.03	**0.06**	0.03	0.05	**0.05**	**0.06**
boat	**0.15**	**0.32**	0.13	0.29	0.10	0.15
bridge	**0.08**	**0.39**	**0.08**	0.36	0.05	0.20
building	**0.04**	**0.41**	**0.04**	0.38	0.03	0.21
bus	**0.26**	**0.68**	**0.26**	0.66	0.22	0.48
caravan	0.27	0.58	**0.31**	**0.64**	0.29	0.45
car	**0.30**	**0.68**	0.29	0.67	0.20	0.59
car-mount	0.29	0.74	0.31	**0.75**	**0.39**	0.73
catch-basin	0.13	0.28	0.15	0.29	**0.26**	**0.34**
cctv-camera	0.07	0.10	0.07	0.10	**0.10**	**0.12**
crosswalk-plain	**0.03**	**0.09**	**0.03**	**0.09**	**0.03**	0.06
curb	**0.09**	**0.33**	**0.09**	0.32	0.07	0.19
curb-cut	**0.04**	**0.11**	**0.04**	0.10	**0.04**	0.07
ego-vehicle	0.24	**0.78**	0.24	0.76	**0.26**	0.55
fence	**0.09**	**0.36**	**0.09**	0.34	0.07	0.20
fire-hydrant	0.23	0.40	0.24	0.40	**0.33**	**0.44**
ground-animal	0.17	0.29	0.17	0.30	**0.26**	**0.33**
guard-rail	**0.12**	**0.44**	**0.12**	0.43	0.11	0.28
junction-box	0.31	0.54	0.32	0.54	**0.43**	**0.58**
lane-marking-crosswalk	**0.05**	**0.14**	**0.05**	0.13	**0.05**	0.09
lane-marking-general	0.09	**0.30**	0.09	0.29	**0.10**	0.23
mailbox	0.22	0.43	0.24	0.45	**0.35**	**0.50**
manhole	0.19	0.39	0.21	0.40	**0.36**	**0.48**
motorcycle	**0.17**	**0.36**	0.16	0.33	0.10	0.15
motorcyclist	0.13	0.24	0.14	**0.25**	**0.15**	0.21
mountain	0.11	**0.38**	**0.12**	0.37	0.10	0.24
on-rails	**0.15**	**0.55**	**0.15**	0.52	0.12	0.30
other-rider	0.17	**0.29**	**0.19**	**0.29**	0.18	0.25
other-vehicle	**0.18**	**0.44**	**0.18**	0.43	0.15	0.27
parking	**0.03**	**0.17**	**0.03**	0.16	**0.03**	0.09
pedestrian-area	**0.06**	**0.53**	**0.06**	0.50	0.05	0.33
person	0.18	**0.33**	**0.19**	**0.33**	**0.19**	0.28
phone-booth	0.26	**0.53**	0.25	0.49	**0.27**	0.40
pole	**0.07**	**0.12**	**0.07**	**0.12**	0.06	0.09
pothole	0.09	0.20	0.10	**0.21**	**0.13**	0.19
rail-track	**0.10**	**0.29**	**0.10**	0.27	0.07	0.15
road	**0.08**	**0.77**	**0.08**	0.74	0.07	0.42
sand	0.18	**0.44**	0.19	**0.44**	**0.21**	0.40
service-lane	0.09	**0.29**	**0.10**	**0.29**	0.07	0.17
sidewalk	**0.08**	**0.42**	0.07	0.40	0.06	0.20
sky	0.12	**0.90**	0.11	**0.90**	**0.17**	0.78
snow	**0.06**	**0.48**	**0.06**	0.47	**0.06**	0.29
street-light	0.12	0.17	0.13	0.18	**0.19**	**0.22**
terrain	0.09	**0.40**	**0.10**	0.39	**0.10**	0.28
traffic-light	0.27	0.40	0.28	0.40	**0.35**	**0.42**
traffic-sign-back	0.20	0.30	0.21	0.30	**0.27**	**0.33**
traffic-sign-frame	**0.09**	**0.24**	**0.09**	0.23	0.07	0.15
traffic-sign-front	0.33	0.52	0.35	0.53	**0.49**	**0.62**
trailer	**0.26**	**0.60**	0.25	0.57	0.21	0.38
trash-can	0.30	0.54	0.31	0.54	**0.41**	**0.56**
truck	**0.27**	**0.67**	**0.27**	0.65	0.25	0.48
tunnel	**0.14**	**0.45**	**0.14**	0.40	0.12	0.26
unlabeled	**0.06**	**0.30**	**0.06**	0.29	**0.06**	0.21
utility-pole	**0.09**	**0.19**	**0.09**	**0.19**	0.08	0.13
vegetation	0.04	**0.39**	**0.05**	0.38	0.04	0.24
wall	0.12	**0.45**	**0.13**	**0.45**	**0.13**	0.34
water	**0.13**	**0.41**	0.12	0.39	**0.13**	0.31
wheeled-slow	**0.16**	**0.31**	**0.16**	0.30	0.14	0.19
**Average**	0.15	**0.39**	0.15	**0.39**	**0.17**	0.30

**Table 6:** A comparison of the SAM’s performance on the validation set of the BDD100K dataset (19 classes) using ViT-H, ViT-L, and ViT-B backbones, evaluated by Mean Intersection over Union (Mean IoU) and Highest Intersection over Union (Highest IoU) for each class. The best performance for each class and metric is highlighted in bold.

	ViT-H		ViT-L		ViT-B	
Class Name	Mean (IoU)	Highest (IoU)	Mean (IoU)	Highest (IoU)	Mean (IoU)	Highest (IoU)
Bicycle	0.04	0.08	0.05	0.10	**0.06**	**0.11**
Building	**0.03**	**0.25**	**0.03**	0.24	**0.03**	0.16
Bus	0.06	**0.18**	**0.07**	**0.18**	**0.07**	0.17
Car	0.07	**0.17**	0.07	**0.17**	**0.08**	0.14
Fence	**0.05**	**0.18**	**0.05**	**0.18**	**0.05**	0.13
Motorcycle	0.05	**0.13**	0.05	**0.13**	**0.06**	0.11
Person	0.05	**0.10**	0.05	**0.10**	**0.06**	0.09
Pole	**0.02**	**0.08**	**0.02**	**0.08**	**0.02**	0.06
Rider	0.04	**0.09**	0.04	**0.09**	**0.05**	**0.09**
Road	**0.05**	**0.45**	**0.05**	0.42	0.04	0.22
Sidewalk	**0.05**	**0.18**	**0.05**	0.17	**0.05**	0.14
Sky	0.06	**0.40**	0.06	**0.40**	**0.07**	0.29
Terrain	**0.05**	**0.16**	**0.05**	**0.16**	**0.05**	0.12
Traffic Light	0.03	**0.08**	0.03	**0.08**	**0.04**	0.07
Traffic Sign	0.05	**0.11**	0.05	**0.11**	**0.07**	0.10
Train	0.03	**0.20**	**0.04**	0.07	0.03	0.05
Truck	**0.07**	**0.20**	**0.07**	0.19	**0.07**	0.13
Vegetation	**0.03**	**0.24**	**0.03**	0.23	**0.03**	0.17
Wall	0.05	**0.18**	0.05	0.17	**0.06**	0.16
**Average**	**0.05**	**0.18**	**0.05**	0.17	**0.05**	0.13

**Table 7:** A comparison of the SAM’s performance on the validation set of the Mapillary Vistas dataset (66 classes) using ViT-H, ViT-L, and ViT-B backbones, evaluated by Mean Intersection over Union (Mean IoU) and Highest Intersection over Union (Highest IoU) for each class. The best performance for each class and metric is highlighted in bold.

	ViT-H		ViT-L		ViT-B	
Class Name	Mean (IoU)	Highest (IoU)	Mean (IoU)	Highest (IoU)	Mean (IoU)	Highest (IoU)
banner	0.28	0.48	0.31	**(IoU)**	**0.36**	**0.51**
barrier	0.13	**0.46**	0.13	0.45	**0.15**	**0.38**
bench	**0.17**	**0.31**	0.14	0.29	0.12	**0.20**
bicycle	**0.14**	**0.28**	0.12	0.27	0.11	**0.19**
bicyclist	**0.18**	**0.33**	**0.18**	**0.33**	0.16	**0.26**
bike-lane	**0.07**	**0.35**	**0.07**	**0.35**	**0.07**	**0.20**
bike-rack	0.09	0.15	**0.11**	**0.16**	0.07	0.12
billboard	0.26	**0.47**	0.26	**0.47**	**0.33**	**0.47**
bird	0.02	0.04	0.02	**0.05**	**0.03**	0.04
boat	0.08	0.22	**0.10**	**0.27**	0.06	0.12
bridge	**0.08**	**0.36**	0.07	0.33	0.05	**0.15**
building	**0.04**	**0.40**	**0.04**	0.38	0.03	**0.21**
bus	**0.25**	**0.68**	0.24	0.65	0.24	**0.51**
car	**0.27**	**0.54**	0.26	0.47	0.25	**0.40**
caravan	0.12	**0.65**	**0.13**	0.64	0.09	**0.55**
car-mount	0.26	0.70	0.28	**0.71**	**0.38**	0.70
catch-basin	0.15	0.31	0.16	0.31	**0.27**	**0.36**
cctv-camera	0.08	0.11	0.08	0.13	**0.14**	**0.15**
crosswalk-plain	**0.04**	**0.11**	0.03	0.10	0.03	**0.06**
curb	**0.09**	**0.34**	**0.09**	0.33	0.07	**0.19**
curb-cut	**0.05**	**0.11**	0.04	**0.11**	**0.05**	**0.09**
ego-vehicle	0.25	**0.77**	0.24	0.75	**0.27**	**0.56**
fence	**0.09**	**0.37**	**0.09**	0.34	0.07	**0.19**
fire-hydrant	0.25	0.39	0.22	0.37	**0.37**	**0.47**
ground-animal	0.22	**0.42**	0.20	0.34	**0.24**	**0.35**
guard-rail	**0.11**	**0.40**	**0.11**	0.39	**0.11**	**0.26**
junction-box	0.29	0.52	0.30	0.53	**0.41**	**0.56**
lane-marking-crosswalk	**0.05**	**0.14**	**0.05**	**0.14**	**0.05**	**0.09**
lane-marking-general	0.09	**0.30**	0.09	**0.30**	**0.10**	**0.24**
mailbox	0.26	0.48	0.28	0.47	**0.39**	**0.50**
manhole	0.20	0.43	0.21	0.42	**0.36**	**0.50**
motorcycle	0.13	**0.33**	**0.14**	0.32	0.08	**0.13**
motorcyclist	0.12	**0.25**	**0.13**	0.24	0.12	**0.18**
mountain	**0.13**	**0.43**	0.12	0.41	0.12	**0.29**
on-rails	**0.17**	**0.60**	0.14	0.51	**0.17**	**0.31**
other-rider	**0.16**	**0.24**	0.15	0.21	0.14	**0.14**
other-vehicle	**0.19**	**0.43**	0.18	0.37	0.13	**0.21**
parking	**0.04**	**0.18**	**0.04**	0.16	0.03	**0.09**
pedestrian-area	0.06	**0.56**	0.06	0.55	**0.07**	**0.43**
person	**0.18**	**0.34**	**0.18**	0.33	0.17	**0.26**
phone-booth	0.21	0.45	0.27	**0.53**	**0.28**	0.42
pole	**0.06**	**0.14**	**0.06**	**0.14**	**0.06**	**0.10**
pothole	0.08	0.20	0.09	0.21	**0.16**	**0.22**
rail-track	**0.12**	**0.29**	0.10	0.27	0.10	**0.17**
road	**0.08**	**0.76**	**0.08**	0.74	0.07	**0.41**
sand	0.20	0.53	**0.25**	**0.56**	**0.25**	0.39
service-lane	0.09	0.30	**0.10**	**0.31**	0.08	0.20
sidewalk	**0.08**	**0.43**	**0.08**	0.40	0.06	**0.22**
sky	0.11	**0.90**	0.11	**0.90**	**0.16**	**0.77**
snow	**0.06**	**0.54**	**0.06**	0.52	**0.06**	**0.33**
street-light	0.11	0.17	0.11	0.18	**0.16**	**0.20**
terrain	**0.09**	**0.40**	**0.09**	0.38	**0.09**	**0.29**
traffic-light	0.24	**0.37**	0.24	0.36	**0.29**	**0.36**
traffic-sign-back	0.19	0.30	0.20	**0.31**	**0.24**	**0.31**
traffic-sign-frame	**0.09**	**0.27**	**0.09**	0.26	0.07	**0.14**
traffic-sign-front	0.30	0.51	0.31	0.52	**0.42**	**0.57**
trailer	0.17	0.53	**0.18**	**0.55**	0.15	0.35
trash-can	0.28	0.53	0.29	0.53	**0.37**	**0.54**
truck	**0.26**	**0.66**	0.25	0.65	0.22	**0.44**
tunnel	0.23	0.53	**0.24**	**0.54**	0.22	0.43
unlabeled	0.06	**0.31**	0.06	0.30	**0.07**	**0.22**
utility-pole	**0.08**	**0.24**	**0.08**	0.20	0.07	**0.12**
vegetation	**0.04**	**0.39**	**0.04**	0.38	**0.04**	**0.23**
wall	0.11	**0.44**	**0.13**	**0.44**	**0.13**	**0.33**
water	**0.16**	**0.46**	**0.16**	0.45	0.14	**0.38**
wheeled-slow	**0.17**	0.29	**0.17**	**0.30**	0.13	0.19
**Average**	0.14	**0.39**	0.15	0.38	**0.16**	0.30

**Table 8:** Inference time and segmentation quality trade-off for 100 random selected images.

Datasets	ViT Backbones	Average No. of Predicted Masks	Average Inference Time (s)	Mean IoU
	ViT-H	59	6.97	**0.10**
KITTI	ViT-L	59	5.61	**0.10**
	ViT-B	30	4.80	0.08
	ViT-H	88	8.45	0.12
BDD100K	ViT-L	87	6.95	**0.13**
	ViT-B	50	5.30	0.12
	ViT-H	94	7.68	0.14
Mapillary Vistas	ViT-L	91	6.47	**0.15**
	ViT-B	51	5.72	0.12

**Table 9:** SAM’s performance in road object segmentation based on road object size.

Datasets	ViT	Avg. IoU for Small Object	Avg. IoU for Medium Object	Avg. IoU for Large Object
KITTI	ViT-H	0.12	0.48	0.64
	ViT-L	0.12	0.48	0.61
	ViT-B	0.05	0.36	0.41
BDD100K	ViT-H	0.18	0.57	0.64
	ViT-L	0.19	0.57	0.60
	ViT-B	0.12	0.47	0.43
Mapillary Vistas	ViT-H	0.01	0.28	0.59
	ViT-L	0.01	0.28	0.58
	ViT-B	0.01	0.27	0.42

**Table 10:** Segmentation performance evaluation of the SAM on the KITTI dataset: Precision, Recall, F1 Score, and True Positive (TP), False Positive (FP), and False Negative (FN).

ViT	Threshold	Precision	Recall	F1 Score	TP	FP	FN
	0.7	0.29	0.57	0.34	1396	1840	851
ViT-H	0.5	0.39	0.64	0.45	1702	1534	851
	0.2	0.51	0.68	0.56	2140	1096	851
	0.7	0.32	0.53	0.34	1322	1849	916
ViT-L	0.5	0.41	0.60	0.44	1623	1548	916
	0.2	0.53	0.64	0.55	2073	1098	916
	0.7	0.14	0.27	0.15	754	1918	1415
ViT-B	0.5	0.19	0.34	0.21	932	1740	1415
	0.2	0.34	0.43	0.34	1281	1391	1415

**Table 11:** Segmentation performance evaluation of the SAM on the BDD100K dataset: Precision, Recall, F1 Score, and True Positive (TP), False Positive (FP), and False Negative (FN).

ViT	Threshold	Precision	Recall	F1 Score	TP	FP	FN
	0.7	0.36	0.78	0.45	59,222	67,944	20,653
ViT-H	0.5	0.50	0.84	0.60	71,909	55,257	20,653
	0.2	0.70	0.87	0.77	92,037	35,129	20,653
	0.7	0.34	0.77	0.44	56,951	70,236	20,632
ViT-L	0.5	0.48	0.84	0.58	69,440	57,747	20,632
	0.2	0.69	0.87	0.76	90,120	37,067	20,632
	0.7	0.25	0.57	0.31	39,488	71,465	36,866
ViT-B	0.5	0.35	0.66	0.42	47,303	63,650	36,866
	0.2	0.53	0.73	0.59	63,029	47,924	36,866

**Table 12:** Segmentation performance evaluation of the SAM on the Mapillary Vistas: Precision, Recall, F1 Score, and True Positive (TP), False Positive (FP), and False Negative (FN).

ViT	Threshold	Precision	Recall	F1 Score	TP	FP	FN
	0.7	0.32	0.56	0.37	228,491	515,928	381,513
ViT-H	0.5	0.40	0.61	0.46	275,049	469,370	381,513
	0.2	0.53	0.66	0.57	348,360	396,059	381,513
	0.7	0.31	0.53	0.36	220,789	497,882	407,261
ViT-L	0.5	0.39	0.59	0.44	265,023	453,648	407,261
	0.2	0.53	0.64	0.56	336,453	382,218	407,261
	0.7	0.24	0.31	0.23	144,438	382,629	598,865
ViT-B	0.5	0.30	0.36	0.28	167,901	359,166	598,865
	0.2	0.41	0.43	0.38	215,463	311,604	598,865

## Data Availability

For this research, the datasets used are publicly available and have been referenced within the main body of the article. (All relevant dataset links are included directly in the corresponding sections of the manuscript.).
